# Community support and promoting cognitive function for the elderly

**DOI:** 10.3389/fpsyg.2022.942474

**Published:** 2022-09-06

**Authors:** Chong Zhang, Daisheng Tang, Yan Wang, Shilin Jiang, Xin Liu

**Affiliations:** ^1^School of Marxism, University of Electronic Science and Technology of China, Chengdu, Sichuan, China; ^2^Beijing Laboratory of National Economic Security Early-Warning Engineering, School of Economics and Management, Beijing Jiaotong University, Beijing, China; ^3^School of Law and Sociology, Xihua University, Chengdu, Sichuan, China

**Keywords:** community support, elderly, cognitive function, social participation, mediating effect

## Abstract

Proper cognitive functions are critical to the life of the elderly. With the rapid aging of the population, community support plays an important role in cognitive functioning. This study examines the association between community support and the level of cognitive functioning in the elderly, and the mediating effect of social participation in the relationship. Based on the panel data of China Longitudinal Healthy Longevity Survey (CLHLS) in 2005, 2008, 2011, 2014, and 2018, people aged 65 and over are selected as the research object (*N* = 35,479). The panel Logit model is used to analyze the influence of community support on their cognitive functioning. In addition, the stepwise regression and KHB decomposition methods are used to test the influence mechanism of community support on their cognitive function. The benchmark regression results show that there is a significant correlation between community support and cognitive function in the elderly (OR: 1.64, 95% CI: 1.41–1.91, *p* < 0.01). Daily care (OR: 1.75, 95% CI: 1.33–2.29, *p* < 0.01) has the strongest impact on the cognitive function of the elderly, followed by health care (OR: 1.70, 95% CI: 1.43–2.01, *p* < 0.01) and legal support (OR: 1.64, 95% CI: 1.37–1.95, *p* < 0.01), while psychological care (OR: 1.62, 95% CI: 1.31–2.01, *p* < 0.01) has the weakest impact on the cognitive function of the elderly. The results of the mediation effect test show that social participation plays a significant intermediary role in the impact of community support on the cognitive function of the elderly (mediation percentage: 16.89%), demonstrating that community support can improve the cognitive function of the elderly by promoting the social participation of the elderly. In classified community support, social participation plays a significant intermediary role in the impact of psychological care on cognition (mediation percentage: 46.10%).

## Introduction

According to data from China’s National Statistical Yearbook, by the end of 2020, approximately 190 million Chinese people are over 65, accounting for 13.50% of the total population. Against the two-fold background of a rapid aging population and the global spread of the Corona Virus Disease 2019 (COVID-19), elderly people in poor health are particularly vulnerable to the virus, and their health is facing unprecedented threats and challenges. Some even face a higher mortality risk. Finding the best way to protect the health of the elderly in this environment of risk and uncertainty has become the focus and an issue deserving social attention. As a significant component of health, cognitive function has also attracted much attention. The survey data from the Chinese Longitudinal Health Longevity Survey (CLHLS) in 2018 demonstrates that the prevalence of cognitive impairment in the elderly in China is 10.4%. According to this ratio, it can be concluded that more than 14 million older people are suffering from cognitive impairment. Severe cognitive impairment is clinically diagnosed as dementia (Hugo and Ganguli, 2014). At the same time, with the accelerated aging of the population, there are increasingly more empty nesters and those living alone (Murayama et al., 2012). While traditional family care functions are gradually weakening (Ng et al., 2002), an increasing number of the elderly tend to choose the mode of community home-based care. Community home-based care helps to increase their health care opportunities and communication and unity among community members, which, in turn, drives healthy aging (Shrestha et al., 2021). The key to community home-based care is to establish an effective community support system that has a certain impact on their cognitive function. Therefore, based on the five-period data of CLHLS, this paper will further study the impact of community support on the cognitive function of the elderly and explore its impact mechanism.

## Literature review

Community support can also be called community service (originally called domestic assistant service) which mainly provides simple personal care, meal rotation, and home cleaning services. Since 2000, this service has been generally divided into two services: enhanced home and community care service (EHCCS) and integrated home care service (IHCS; Lai et al., 2009). However, community support has been recognized as a multidimensional concept, such as daily living support like preparing meals and bathing (LaVeist et al., 1997), emotional and spiritual support, health care support, and so on (Liu et al., 2016). Therefore, when discussing community support, we should consider various structures of community support. In the present study, we focus on four dimensions of community support, namely health care, daily care, psychological care, and legal support.

As an important element of health, cognition has always been taken into account by the academic circles. Cognitive functions can be defined as memory, thinking, reasoning, problem-solving, planning, and processing speed and are also broadly described as aspects of human intelligence (Shuyang and Meng, 2021). Cognitive impairment refers to the impairment of one or more of the above cognitive functions, which affects the ability of memory, learning, and decision-making in personal daily life (An and Liu, 2016).

Previous studies have shown that community support has a positive effect on cognitive function of the elderly; in particular, functional support and emotional community support are very important to the cognitive function of the elderly (Hiroshi et al., 2019; Souza-Talarico et al., 2021). Functional community support is mainly reflected in medical service, community infrastructure construction, community environment construction, and so on. For example, community medical service can better alleviate the decline of cognitive function in the elderly (Zhu et al., 2012), especially for those in poor health and community support can promote their cognitive function (Foong et al., 2021). In addition, the community environment also affects their cognitive function (Xiang et al., 2018). There is a positive correlation between community infrastructure and cognitive function, especially health and entertainment facilities (Yue et al., 2021). Emotional community support is mainly transferred through the processing of family relations and neighborhood relations, as well as the expansion of social networks. Studies have shown that support from family and friends is crucial to proper cognitive functions in the elderly (Ge et al., 2017; Noguchi et al., 2019; Pais et al., 2021), while some studies have shown that there is no direct relationship between family support and cognitive function (Li et al., 2019). The enhancement of social networks can reduce their depression and self-isolation, which is conducive to their cognitive health (Siette et al., 2020). Conversely, some scholars have found that there is no evident relationship between social networks and elderly cognition (Vance et al., 2016).

From the perspective of activity theory, active participation in social activities can help the elderly maintain their living conditions, role functionality, and interpersonal relationships. As in their youth, they can find meaning in life, and obtain a sense of belonging, satisfaction, and happiness, thereby alleviating the social role interruptions caused by depression and cognitive deterioration. Community support can protect the legitimate rights and interests when they are engaged in social activities. At the same time, the community organizes cultural, sports, and recreational activities to improve their level of social participation of the elderly. Studies have shown that community support can promote social participation for the elderly (Ho et al., 2021; Siette et al., 2021), In addition, social participation can effectively slow the decline of cognitive function in the elderly (House et al., 1988; Hughes et al., 2013; Lea et al., 2015; Dause and Kirby, 2019). It can be presumed that social participation may play an intermediary role between community support and cognitive function. At present, no research has confirmed this point. Therefore, this paper selects social participation as an intermediary variable to explore how social support affects cognitive performance in old age.

In addition to community support, the cognitive function of the elderly is also affected by other factors, such as social support, individual education level, lifestyle, and so on. Social support has a positive effect on individual self-identification. Many studies have found that social support can effectively prevent cognitive decline (Yeh and Liu, 2003; Andel et al., 2012; Moreno et al., 2021). For example, emotional social support can ameliorate the negative emotions of the elderly (Seeman et al., 2001), such as depression, anxiety, and other emotional states (Cai et al., 2011), thereby protecting healthy cognitive function. Education can also improve their cognitive abilities (Inouye et al., 1993; Lee et al., 2003). Furthermore, a good lifestyle can slow cognitive decline (Kang et al., 2021), such as physical activity (Lü et al., 2016) and fitness (Daimiel et al., 2020) as well as good nutrition (Smith and Blumenthal, 2016). On the contrary, smoking increases the risk of cognitive impairment in the elderly (Souza-Talarico et al., 2021). In addition, maintaining one’s interests can also help maintain a state of normal cognition (Iwasa et al., 2012).

In summary, previous studies have comprehensively discussed the relationship between community support and elderly cognitive performance, but a majority of them are cross-sectional studies. There are few studies that have integrated four dimensions of health care, daily care, psychological care, and legal support to explore the impact of community support on older adults. This paper will use the five-period longitudinal data from CLHLS to study the impact of various community support on the cognition of the elderly. Taking social participation as the intermediary variable, this paper further discusses the mechanism of community support that impacts the cognitive function of the elderly.

## Data, measurement, and methods

### Data

Research data in this paper has been extracted from the CLHLS, the largest national longitudinal data sample in China organized by the Center for Healthy Ageing and Development at Peking University. The CLHLS-based line survey was conducted in 1998, and subsequent follow-up surveys were conducted in 2000, 2002, 2005, 2008, 2011, 2014, and 2018. The baseline survey and follow-up survey covered 23 of China’s 31 provinces, and about half of the cities and counties were randomly selected as survey sites in the 22 surveyed provinces. In order to ensure the continuity of the follow-up survey and the comparability of different time points, the elderly who died or could not be found for follow-up are to be replaced according to the principle of the same sex and same age. Considering the content of the questionnaire and the representativeness of the sample, this study selects five-period data from CLHLS in 2005, 2008, 2011, 2014, and 2018. After removing some irrelevant data and missing samples, the effective sample sizes in 2005, 2008, 2011, 2014, and 2018 are 10,492, 9,461, 5,289, 3,636, and 6,601, respectively.

### Measurement

Dependent variable: The target variable is cognitive function. We obtain the variable from the item in the CLHLS. This questionnaire is based on the Mini-Mental State Examination (MMSE) and modified according to China’s national conditions. The reading and writing ability test items were deleted, while “The number of foods in a minute” was added. The revised scale contains five dimensions and a total of 24 questions to gauge general ability, reaction ability, attention and calculation ability, memory and language, comprehension, coordination, and self-coordination ability. Except for the item “Say the number of foods in a minute” which counts for seven points (one point for each item of food, seven points for seven or more items), all other items are worth one point for a total of 30 points. The scale presents good reliability in the effective sample size from the five-period data. The internal consistency coefficients Cronbach α in 2005, 2008, 2011, 2014, and 2018 are 0.850, 0.855, 0.806, 0.780, and 0.784, respectively. The effective aggregate sample size of these for surveys also has high reliability, and the Cronbach α values is 0.839. The KMO value of the structural validity analysis is also very sound. Its values in 2005, 2008, 2011, 2014, and 2018 are 0.929, 0.919, 0.902, 0.881, and 0.898, respectively. The KMO value of the effective summary sample size of the five surveys reached 0.922. Combined with previous related research contained in the CLHLS, it can be defined as normal cognitive function and coded as 1, when the cognitive function item scores no less than 24 points. If not, it will be defined as impaired cognitive function, coded as 0 (Zeng and Vaupel, 2002; Zeng 2013).

Explanatory variables: The primary explanatory variable is community support. We obtain the variable from the CLHLS item (What kind of social services are available in your community?), involving personal daily care services, home visits, psychological consulting, daily shopping, social and recreation activities, legal aid, health education, and neighboring relations. The options are “Yes” and “No.” If the respondent chooses “No” to the above eight items, it means there is no community support (coded as 0); those who choose one or more of them are thought of as having community support (coded as 1). At the same time, considering the differences among various types of community support, this research subdivides community support into four categories: health care, daily care, psychological care, and legal support. Health care involves home visits and health education. If none of these two items are available, health care is coded as 0. If one or two of them are presented, it is coded as 1. The latter three types of services are treated similarly. Daily care includes personal daily care services and daily shopping; psychological care includes psychological consulting and social and recreation activities. Legal support includes legal aid and neighboring relations.

Intermediary variable: Social participation. We select the following three activities to operationalize social participation, namely “Are you currently engaged in activities such as playing cards or mahjong?,” “Are you currently participating in social activities (organized activities)?” and “How many tours have you traveled on during the year?.” According to the responses, people who did not engage in one of these three activities is defined as without social participation, coded as 0. Those who have engaged in one or more is defined as having social participation, coded as 1.

Control variables: Referring to the previous literature on the factors affecting the cognitive function of the elderly, in addition to community support, control variables that may affect cognitive function in the elderly were also taken into account, such as gender (male coded as 1, female coded as 0), age (65–117 years old), residence (urban coded as 1, rural area coded as 0), education level (educated coded as 1, illiterate coded as 0), marital status (with spouse coded as 1, without spouse coded as 0), activity of daily living (ADL, fully able to take care of oneself coded as 1, unable to take care of oneself coded as 0), instrumental activities of daily living (IADL, fully able to take care of oneself coded as 1, unable to take care of oneself coded as 0), whether they smoke (yes coded as 1, no coded as 0), whether they drink alcohol (yes coded as 1, no coded as 0), whether they exercise regularly (yes coded as 1, no coded as 0), and sleep quality (The variable is coded as 1, 2, 3, 4, and 5, corresponding to very good, good, general, bad, and very bad). Depression was also studied and measured according to the depression scale in the questionnaire that contains seven questions. The questions covered whether they are “seeing things positively,” “keeping your things neat and tidy,” “making your own decisions,” “being as happy as you were when you were young,” “feeling scared and anxious,” “feeling lonely,” and “feeling useless as you get older.” The final score ranges from 7 to 35 points. According to previous research experience, a score in the range of 7 to 20 is defined as mild depression and assigned a value of 1; a score in the range of 21 to 35 is defined as severe depression and assigned a value of 0 (Zhang and Lan, 2021). Relative economic level is indicated according to respondents’ self-assessment, the variable is coded as 1, 2, 3, 4, and 5, corresponding to very rich, relatively rich, general, relatively poor, and very poor. Whether the source of livelihood is sufficient if yes coded as 1, and no is coded as 0, etc.

### Methods

#### Panel logit model

Based on the five-period survey data, and considering that both the dependent variable and the independent variable are treated as binary variables, we select the panel binary selection model for research. Commonly used panel binary selection models include the panel Probit model and the panel Logit model. The former can only be estimated by using a random effect model, and cannot effectively control individual effects or (and) time effects. Therefore, the panel Logit model is selected for empirical analysis. The basic model is as follows:


(1) $$P(y_{it}=1\left|x_{it}\right.,\beta ,\mu_{i)}=\Lambda \left(\mu_{i}+x\prime_{it}\beta \right)=\frac{e^{\mu i+x\prime_{it}\beta }}{1+e^{\mu i+x\prime_{it}\beta }}$$

In the equation, $y_{it}$ is the dependent variable and whether the cognitive function is normal; $x_{it}$ is the vector constituted by the independent variable (may include the lag term); β is the coefficient vector; $\mu_{it}$ is the individual effect; Λ(⋅) is the cumulative distribution function of the logistic distribution. The subscript *i* indicates the elderly in order *i*, and the subscript *t* indicates the year in order *t*.

#### Mediation effect

In order to further study the influence mechanism of community support on elder cognitive function, social participation is set as an intermediary variable for analysis. We use the following three equations to estimate the mediation effect (Baron and Kenny, 1986).


(2) $$CF_{i}=a_{1}+\beta_{1}CS_{i}+\lambda_{1}X_{i}+\varepsilon_{i1}$$

(3) $$M_{i}=a_{2}+\beta_{2}CS_{i}+\lambda_{2}X_{i}+\varepsilon_{i2}$$

(4) $$CF_{i}=\alpha_{3}+\beta_{3}CS_{i}+\zeta M_{i}+\lambda_{3}X_{i}+\varepsilon_{i3}$$

Under normal circumstances, if $\beta_{1}$ , $\beta_{2}$ , and ζ are significant, the mediating effect is significant. Through Equation (4), we can also estimate the value of the mediation effect. If $\beta_{3}$ is not significant, it means that the effect of community support on cognitive function is completely mediated by social participation.

## Results

### Characteristics of the sample

**Figure 1:**
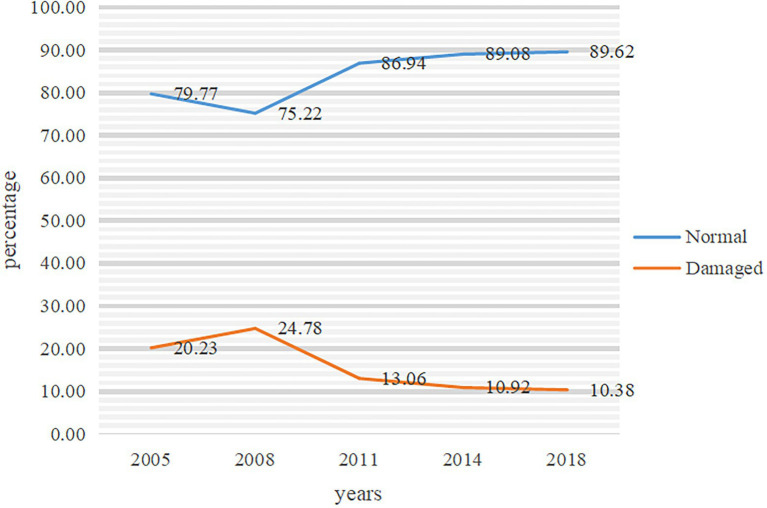
Basic information about cognitive function in the elderly.

According to the five-period survey data, in 2018, the proportion of the elderly with normal cognitive function is the highest, reaching 89.62%, while in 2008, it is the lowest, only 75.22% (Figure 1). However, it does not indicate that the cognitive function of the elderly has a tendency to improve. As shown in Table 1, from the 2005 wave to the 2018 wave, the average ages of the effective samples of the elderly are 82.92, 83.84, 82.25, 82.38, and 80.91, respectively.

**Table 1:** Sample characteristics (five periods pooled data).

Variables	2005	2008	2011	2014	2018
Community support, %	33.43	26.24	50.52	62.51	64.98
Social participation, %	36.11	29.70	36.89	38.53	40.39
Gender, %	48.53	48.59	51.75	51.76	49.55
Age, mean (SD)	82.92 (11.16)	83.84 (10.82)	82.25 (10.28)	82.38 (9.30)	80.91 (10.54)
Residence, %	45.06	39.73	48.78	47.66	59.29
Education level, %	45.53	44.47	51.62	51.84	63.69
Marital status, %	40.17	40.30	48.18	48.13	53.07
ADL, %	86.74	89.80	86.27	87.84	88.02
IADL, %	42.44	42.10	47.46	49.53	47.08
Smoke, %	39.74	37.43	38.72	33.58	33.04
Drink, %	36.57	33.54	34.83	28.27	28.47
Exercise, %	49.80	45.25	50.95	37.90	45.48
Sleep quality, %					
Very good	15.18	13.24	19.87	17.05	16.32
Good	51.21	52.70	44.00	46.78	37.54
General	24.10	24.50	24.33	25.63	31.83
Bad	8.81	8.91	10.78	9.63	12.3
Very bad	0.70	0.64	1.02	0.91	2.01
Depression, %	12.87	15.85	9.59	9.90	8.47
Relative economic level, %					
Very rich	1.46	1.09	1.51	1.84	2.98
Relatively rich	16.27	13.16	17.89	16.83	17.89
General	67.55	68.65	67.65	72.00	70.29
Relatively poor	12.42	14.57	10.72	8.06	7.82
Very poor	2.31	2.54	2.23	1.27	1.01
Sufficient source of livelihood, %	79.34	77.92	81.87	84.24	87.59
*N*	10,492	9,461	5,289	3,636	6,601

The sample characteristics are shown in Table 1. It is worth noting that the overall proportion of elderly people with community support and social participation is not cause for optimism, but since 2008, the proportion has increased at each survey time point, which is roughly the same trend as the proportion of normal cognitive function in Figure 1.

### Panel logit regression results

#### The overall impact of community support on the cognitive function of the elderly

In order to compare the results of different models, Table 2 presents the results of the pooled regression model, fixed-effects model, and random-effects model. The pooled regression model assumes that there is no individual effect, and aggregates the data at all-time points for regression analysis, ignoring the missed heterogeneity among individuals. Additionally, the possible association between this heterogeneity and explanatory variables will lead to an estimated deviation. Although the random effects model can solve the problem of estimation bias caused by missing variables to a certain extent, this strategy must assume that the missing variables will not affect the explanatory variables. Once the missing variables are correlated with the explanatory variables, it may be led to biased analysis results. The fixed effect is to fix the individual differences at different time points, thus effectively eliminating the influence of unobserved omitted variables on the dependent variable and the interference effect on the relationship between the independent variable and the dependent variable (Li and Liu, 2015). From the panel estimation results of the influence of community support on the cognitive function of the elderly, the Hausman test shows that the fixed effect is the optimal model. As shown in Table 2, community support has a significant positive impact on their cognitive function, demonstrating that those who have accessed community support are more likely to have normal cognitive function (OR: 1.64, 95% CI: 1.41–1.91, *p* < 0.01). We also observe that age has a significant negative impact on cognitive function. The older they are, the lower the possibility of normal cognitive functioning. Older people in urban areas are more likely to have normal cognitive function. In addition, ADL and IADL would be strong predictors of sustained elderly cognitive function. Those who exercise regularly are also more likely to have normal cognitive function. Better sleep quality can also increase the possibility of maintaining normal cognitive function. Elderly people with less depression or sufficient sources of livelihood are also more likely to have normal cognitive function.

**Table 2:** Panel estimation results of the impact of community support on the cognitive function of the elderly, OR (95% CI).

Variables	Pooled effects	Fixed effects	Random effects
Community support	1.40 (1.30–1.51)^***^	1.64 (1.41–1.91)^***^	1.40 (1.30–1.51)^***^
Gender	1.47 (1.33–1.61)^***^	1.74 (0.44–6.92)	1.47 (1.33–1.61)^***^
Age	0.94 (0.93–0.94)^***^	0.97 (0.94–0.99)^***^	0.94 (0.93–0.94)^***^
Residence	1.29 (1.20–1.39)^***^	1.25 (1.05–1.48)^**^	1.29 (1.20–1.39)^***^
Education level	2.15 (1.97–2.36)^***^	1.05 (0.57–1.94)	2.15 (1.97–2.36)^***^
Marital status	1.21 (1.10–1.32)^***^	1.04 (0.79–1.36)	1.21 (1.10–1.32)^***^
ADL	1.91 (1.74–2.10)^***^	1.66 (1.33–2.07)^***^	1.91 (1.74–2.10)^***^
IADL	2.80 (2.54–3.08)^***^	2.26 (1.91–2.69)^***^	2.80 (2.54–3.08)^***^
Smoke	0.92 (0.83–1.00)^*^	0.76 (0.54–1.07)	0.92 (0.83–1.00)^*^
Drink	0.89 (0.81–0.97)^***^	0.93 (0.68–1.26)	0.89 (0.81–0.97)^***^
Exercise	1.26 (1.17–1.36)^***^	1.19 (0.99–1.43)^*^	1.26 (1.17–1.36)^***^
Sleep quality	0.98 (0.94–1.02)	0.92 (0.84–1.00)^*^	0.98 (0.94–1.02)
Depression	0.51 (0.46–0.56)^***^	0.69 (0.56–0.84)^***^	0.51 (0.46–0.56)^***^
Relative economic level	0.86 (0.80–0.91)^***^	0.96 (0.85–1.09)	0.86 (0.80–0.91)^***^
Sufficient source of livelihood	1.36 (1.24–1.50)^***^	1.39 (1.15–1.69)^***^	1.36 (1.24–1.50)^***^
_constant	360.48 (212.89–610.39)^***^	—	360.48 (212.89–610.39)^***^
Hausman test	chi2(15) = 80.91, Prob>chi2 = 0.000		
		chi2(15) = 80.91, Prob>chi2 = 0.000	

* *p < 0.1;*

** *p < 0.05;*

*** *p < 0.01*.

#### The impact of classified community support on the cognitive function of the elderly

From the panel estimation results of the impact of classified community support on the cognitive function of the elderly, the Hausman test shows that the fixed effects are also optimal models. Due to the limited space, only the fixed effects regression results are retained here. From the estimation results (Table 3), the four types of community support: health care (OR: 1.70, 95% CI: 1.43–2.01, *p* < 0.01), daily care (OR: 1.75, 95% CI: 1.33–2.29, *p* < 0.01), psychological care (OR: 1.62, 95% CI: 1.31–2.01, *p* < 0.01), and legal support (OR: 1.64, 95% CI: 1.37–1.95, *p* < 0.01) all have significant positive impacts on cognitive function. Among them, daily care has the strongest influence on cognitive function, and psychological care has the weakest influence on the cognitive function. In addition to community support, age, residence, ADL, IADL, exercise, sleep quality, depression, and sufficient sources of livelihood still have significant impacts on the cognitive function.

**Table 3:** Panel estimation results of the impact of classified community support on the cognitive function of the elderly, OR (95% CI).

Variables	Fixed effects			
Health care	1.70 (1.43–2.01)^***^	—	—	—
Daily care	—	1.75 (1.33–2.29)^***^	—	—
Psychological care	—	—	1.62 (1.31–2.01)^***^	—
Legal support	—	—	—	1.64 (1.37–1.95)^***^
Gender	1.70 (0.43–6.76)	1.66 (0.42–6.55)	1.75 (0.44–7.01)	1.85 (0.45–7.57)
Age	0.96 (0.94–0.99)^***^	0.98 (0.96–1.00)	0.98 (0.96–1.00)^*^	0.98 (0.96–1.00)^*^
Residence	1.25 (1.05–1.48)^**^	1.25 (1.05–1.48)^**^	1.24 (1.05–1.48)^**^	1.23 (1.04–1.47)^**^
Education level	1.05 (0.57–1.93)	0.10 (0.54–1.83)	1.02 (0.55–1.88)	1.07 (0.58–1.98)
Marital status	1.06 (0.81–1.39)	1.02 (0.78–1.34)	1.02 (0.78–1.34)	1.03 (0.79–1.35)
ADL	1.68 (1.34–2.10)^***^	1.63 (1.31–2.04)^***^	1.63 (1.30–2.03)^***^	1.62 (1.30–2.02)^***^
IADL	2.24 (1.89–2.65)^***^	2.23 (1.88–2.65)^***^	2.23 (1.88–2.64)^***^	2.23 (1.88–2.65)^***^
Smoke	0.79 (0.57–1.11)	0.80 (0.57–1.12)	0.78 (0.56–1.09)	0.80 (0.57–1.12)
Drink	0.92 (0.68–1.26)	0.94 (0.69–1.28)	0.93 (0.68–1.26)	0.93 (0.68–1.26)
Exercise	1.17 (0.98–1.40)^*^	1.16 (0.97–1.39)^*^	1.17 (0.98–1.40)^*^	1.20 (1.00–1.44)^**^
Sleep quality	0.91 (0.84–1.00)^**^	0.91 (0.83–0.99)^**^	0.92 (0.84–1.00)^**^	0.91 (0.84–1.00)^**^
Depression	0.68 (0.56–0.83)^***^	0.69 (0.56–0.84)^***^	0.70 (0.57–0.85)^***^	0.68 (0.56–0.83)^***^
Relative economic level	0.97 (0.85–1.10)	0.96 (0.84–1.09)	0.95 (0.84–1.08)	0.96 (0.84–1.09)
Sufficient source of livelihood	1.36 (1.12–1.65)^***^	1.37 (1.13–1.66)^***^	1.34 (1.11–1.63)^***^	1.37 (1.13–1.66)^***^

* *p < 0.1;*

** *p < 0.05;*

*** *p < 0.01*.

### Mediating effect analysis

In this study, the mediating effect of social participation is verified by the stepwise regression method. Under the premise of controlling other variables, model 1 tested the effect of independent variable community support on cognitive function in the elderly. The results show that community support has a significant positive effect on cognitive function. Model 2 examines the impact of community support on the mediating variable of social participation, and the results show that community support has a significantly positive impact on social participation (OR: 1.88, 95% CI: 1.77–2.01, *p* < 0.01). Model 3 examines the impact of community support (OR: 1.34, 95% CI: 1.24–1.44, *p* < 0.01) and social participation (OR: 1.80, 95% CI: 1.64–1.97, *p* < 0.01) on cognitive function (Table 4). The results show that these two still have significantly positive impacts on the cognitive function of the elderly, and the coefficient of community support is significantly reduced. According to the criteria for testing the mediating effect, it can be preliminarily determined that social participation plays a partial mediating role in the impact of community support on cognitive function, and according to the decomposition results of the KHB (Karlson et al., 2012) mediating effect, the mediation percentage is 16.89%.

**Table 4:** Mediating effect stepwise regression results, OR (95% CI).

	Cognitive function	Social participation	Cognitive function
	model 1	model 2	model 3
Community support	1.40 (1.30–1.51)^***^	1.88 (1.77–2.01)^***^	1.34 (1.24–1.44)^***^
Social participation			1.80 (1.64–1.97)^***^

*** *p < 0.01*.

The mediating effects of social participation on different types of community support for elderly cognitive function are further analyzed by using the KHB mediation effect decomposition method. The estimation results are shown in Table 5. The results show that social participation does not play mediating roles in the impact of health care (OR: 1.01, 95% CI: 1.00–1.03, *p* > 0.1), daily care (OR: 1.00, 95% CI: 0.99–1.02, *p* > 0.1), and legal aid (OR: 1.01, 95% CI: 0.99–1.03, *p* > 0.1) on cognitive function, but plays an important mediating role in the impact of psychological care (OR: 1.09, 95% CI: 1.07–1.11, *p* < 0.01) on cognitive function. According to the decomposition results of the KHB mediating effect, it can be seen that the mediating effect of social participation in the process of psychological care affecting cognitive function in the elderly is 46.10%.

**Table 5:** Regression results of KHB mediation effect, OR (95% CI).

	Total Effect	Direct Effect	Indirect Effect
Health care- Social participation- Cognitive function	1.51 (1.37–1.66)^***^	1.49 (1.36–1.64)^***^	1.01 (1.00–1.03)
Daily care- Social participation- Cognitive function	0.89 (0.78–1.02)^*^	0.89 (0.78–1.02)^*^	1.00 (0.99–1.02)
Psychological care- Social participation- Cognitive function	1.21 (1.08–1.35)^***^	1.11 (0.99–1.24)^*^	1.09 (1.07–1.11)^***^
Legal support- Social participation- Cognitive function	1.03 (0.93–1.14)	1.02 (0.92–1.12)	1.01 (0.99–1.03)

* *p < 0.1* and

*** *p < 0.01*.

## Discussion

Based on the five-period data of CLHLS in 2005, 2008, 2011, 2014, and 2018, the panel Logit model is used to analyze the impact of community support on elderly cognitive function. The research results of the existing literature show that there are disputes about the impact of community support on cognitive function. There are mainly two views, one is that community support has a positive effect on elderly cognitive function and the other is that community support has no relationship with the cognitive function. The results of this study show that community support has a significant positive impact on the cognitive function of the elderly, that is, the elderly with community support are more likely to have normal cognitive function. The reason may be that community support can provide various material and spiritual services for the elderly, and build a bridge between the elderly and the outside world. The elderly with community support can get a certain amount of compensation both physically and psychologically. As for sustenance, this has had strong defensive and protective effects on cognitive function, and has helped to continuously maintain and strengthen their self-cognition.

From the perspective of community support classification, the four types of community support have significantly positive impacts on cognitive function. Among them, daily care has the strongest impact and psychological care has the weakest impact. The strongest effect of daily care may be due to the fact that daily care can promote mental health to some extent and indirectly improve cognitive function. With the increase of age, elder self-care ability will decline constantly. The community providing daily care can significantly alleviate the sense of helplessness and loneliness, thereby improving their cognitive function. The weakest impact of psychological care may be due to some deficiencies in the spiritual and cultural aspects of community support construction in China at the present stage, which leads to a weak effect. There are three possible reasons: First, the coverage is small (the supply of psychological care in the five-period data is 16.16, 12.59, 18.76, 24.42, and 29.28%), and participation of the elderly is low. Second, activities are not concentrated, single form, ignoring the physical and psychological individual differences. Third, the organization needs to be strengthened and the effect is not obvious. Only a few have a sense of gain. In addition, legal support also has a great impact on the cognitive function. The reason may be that the protection of the legitimate rights and interests of the elderly and the maintenance of their harmonious relationship with neighbors and family members can reduce the stimulation of negative emotions such as anger, fear, anxiety, and depression on cognitive function to a certain extent. Good neighborhood relations and family relations environment can also promote cognitive function (Ge et al., 2017).

In addition, age is inversely associated with cognitive function in older adults. There is a positive relationship between residence and cognitive function, that is, compared with rural residents, urban residents have better cognitive function, which has also been confirmed by many previous studies (Saenz et al., 2018; Wang, 2021). The more normal ADL and IADL are, the better is their self-care ability, the less likely they will suffer from cognitive impairment. The elderly who exercise have a lower risk of cognitive impairment, which may be due to the more active state of the brain and body organs and the timely release of mental stress. Appropriate exercise is also a way to enhance physique and delay aging. The higher the sleep quality, the higher the possibility of normal cognitive function and mental state which has also been confirmed in previous studies (Mousavi et al., 2020). The higher the degree of depression is, the weaker the cognitive function is. The better the relative economic level is, the higher the possibility of normal cognitive function. This is because the elderly with higher relative economic level often have more time and energy to pay attention to their own health and are willing to invest more money in health. Compared to those with insufficient source of livelihood, the elderly with sufficient source perform better on cognitive function tests, which may due to relatively little financial pressure and a more relaxed and free mentality.

In the last part of the empirical analysis, this study also confirms that social participation plays a mediating role in the impact of community support on elder cognitive function, but there are significant differences in the proportion of social participation mediators in different areas of community support. Specifically, the mediation of social participation accounts for 16.89%, When psychological care is taken as an independent variable, the mediation of social participation accounts for 46.10%. When health care, daily care, and legal support are taken as independent variables, there is no mediating effect of social participation. The above research results can be interpreted from the following two perspectives: community support can promote the social participation of the elderly (Jin and Zhang, 2017). The improvement of social participation has a significant role in promoting cognitive function, provides spiritual sustenance, reduces loneliness, and raises their sense of achievement, which helps them gain a sense of self-fulfilling happiness (Chen, 2022). On the other hand, because the elderly have psychological needs to be respected, cared for, and their self-worth realized (Yao et al., 2020), psychological care can satisfy these needs, promote social participation, and improve their cognitive function.

## Conclusion

Based on the empirical conclusions of this study, we believe that we should pay close attention to the impact of community support on the cognitive levels of the elderly, expand community support coverage, establish a professional team for providing community services, promote social participation, improve cognitive functions overall, and make positive contributions to active aging. It is worth noting that there are still two limitations in this paper: First, the community support used is a service provided by the community, which may be different from the actual needs and usage of the elderly. Therefore, the impact of the actual use of community services on elderly cognition needs to be studied further. Second, limited by the availability of data, the social participation in this paper is only measured by the participation in mahjong, tourism, and social organization activities, and some activities may be omitted. We hope that this study will encourage further research on the interrelationship between community support and the cognitive health of the elderly, and additionally, the role of social participation in the process of community support related to enhancing elder cognition.

## Data availability statement

Publicly available datasets were analyzed in this study. This data can be found at: https://opendata.pku.edu.cn/dataset.xhtml?persistentId=doi:10.18170/DVN/WBO7LK.

## Ethics statement

The studies involving human participants were reviewed and approved by the Center for Healthy Aging and Development Studies (CHADS) at Peking University. Written informed consent for participation was not required for this study in accordance with the national legislation and the institutional requirements. Ethical review and approval was not required for the study on human participants in accordance with the local legislations and institutional requirements. Written informed consent was obtained from all participants for their participation in this study. Written informed consent was obtained from the individual(s) for the publication of any potentially identifiable images or data included in this article.

## Author contributions

CZ is responsible for the empirical analysis and full text drafting. DT is responsible for the conclusion and article revision. YW is responsible for the data, variables, and methods. SJ is responsible for the literature review. XL is responsible for the introduction. All authors contributed to the article and approved the submitted version.

## Funding

This work was supported by the University of Electronic Science and Technology of China Scientific Research Start-up Fund (Grant no. Y030222059002015). The funding body has no role in the design of the study, data collection, analysis, interpretation of the data, and write-up of the manuscript.

## Conflict of interest

The authors declare that the research was conducted in the absence of any commercial or financial relationships that could be construed as a potential conflict of interest.

## Publisher’s note

All claims expressed in this article are solely those of the authors and do not necessarily represent those of their affiliated organizations, or those of the publisher, the editors and the reviewers. Any product that may be evaluated in this article, or claim that may be made by its manufacturer, is not guaranteed or endorsed by the publisher.
